# Medical services of a mulicultural summer camp event: experiences from the 22nd World Scout Jamboree, Sweden 2011

**DOI:** 10.1186/1472-6963-13-187

**Published:** 2013-05-22

**Authors:** Ib Jammer, Christina Allansdotter Andersson, Anna Lindholm Olinder, Bo Selander, Anna Elmerfeldt Wallinder, Stefan Rocco Hansson

**Affiliations:** 1 Department of Anaesthesia and Intensive Care, Haukeland University Hospital, 5021 Bergen, Norway; 2 Department of Pediatrics, Central Hospital, 291 85 Kristianstad, Sweden; 3 Department of Clinical Science and Education, Karolinska Institute, Södersjukhuset, 118 83 Stockholm, Sweden; 4 Primary Care Centrum, County Council Västernorrland, 852 34 Sundsvall, Sweden; 5 Department of Obstetrics and Gynecology, Institute for Clinical Sciences Lund, Lund University, 221 85 Lund, Sweden

**Keywords:** Health services, Scout, Camp, Morbidity, Adolescent, Health policy, Safety, Paediatrics

## Abstract

**Background:**

Prevention and treatment of medical issues are the main task of a health service at a youth camp. However, only few reports about organisation and implementation of camp health care are available. This makes it difficult for future camp directors to plan and estimate the health care needed for a certain camp size. We summarize the experience in planning and running health care for the 22nd World Scout Jamboree (WSJ) 2011 in Sweden.

**Methods:**

During the WSJ, 40,061 participants from 146 nations were gathered in southern Sweden to a 12 day summer camp. Another 31,645 people were visitors. Members for the medical service were 153 volunteering medical professionals with different language and cultural backgrounds from 18 different countries.

**Results:**

Of 40,061 participants 2,893 (7.3%) needed medical assistance. We found an equal distribution of cases to approximately one third surgical, one third medical and one third unspecified cases. Much energy was spent on health prevention, hygiene measures and organizing of psychological support.

**Conclusions:**

A youth camp with a multicultural population and a size of a small city demands flexible staff with high communication skills. Special attention should be paid in prevention of contagious diseases and taking care of psychological issues.

## Background

When Sir Baden-Powell initiated the scout movement, camping and outdoor experience became an important part in youth education [1]. Camping itself provides a unique platform for youth development that is different from experiences that youth gather at school or at home. The body of research regarding camp experience is expanding since the last decade which has led to an improved understanding of the values in these activities [2]. A camp experience improves positive youth development including self-esteem, peer relationships, independence, leadership and social competence [3]. The connection with the natural environment has been associated with positive impact on physical and psychological conditions like depression, attention disorder and obesity [4].

Sir Baden-Powell stated in his original ”Scouting for boys” that without basic skills in first aid the scouts might as well stay home [1]. Today, it is a requirement that sufficient health service is provided at larger camp sites. Health service should be provided at camps, especially in the field where participants are disabled or have special needs [5,6]. Outbreaks of infectious diseases during camps are related to the amount of people living together in provisional housing or by environmental exposure to a pathogen [7,8].

Health prevention on camps was adopted early in scouting by its founder Sir Baden-Powell [9]. Prevention as well as treatment of medical issues should be the main task of the health service when planning a camp [10,11]. Hygiene is a key word emphasized by Sir Baden-Powell. He emphasized in his book the importance to keep the camp, the camp kitchen and yourself clean to avoid becoming sick [1]. However, only few reports about camp health care are available regarding the organisation and implementation of health care at big camps [12,13]. This makes it difficult for future camp directors to plan and estimate the health care needed for a certain camp size.

In this article we summarize the experiences in planning and running health care for the 22^nd^ World Scout Jamboree (WSJ) 2011 in Sweden. During the WSJ, 40,061 participants from 146 nations were gathered together with additional 31,645 visitors during the event. This makes the World Scout Jamboree in Sweden 2011 the largest World Scout Jamboree ever held, so far.

## Methods

The WSJ is an international scout camp of the World Organisation of the Scout Movement. It takes place every fourth year, the location changes each time and rotates through the continents. National Scouting Organisations apply to arrange and host the jamboree. In 2011, the Scout Council of Sweden hosted the WSJ from July 27th to August 7th 2011 in Rinkaby, a military training field outside Kristianstad in Sweden. The area of the campsite was approximately 2 km^2^. A total 40,061 scouts from 146 countries took part in the camp. The target group was adolescents between 14 and 17 years. One quarter of the participants were scout leaders or service staff 18 years and older. The total number of participants was equivalent to the number of inhabitants in an average Swedish city. The camp demanded therefore a sophisticated infrastructure for transportation, food delivery, waste handling, water and drainage as well as health care. All work during the planning phase and the executive phase of the Jamboree was performed by volunteers. The camp site had access to drinking water and showers. Approximately 1,200 flush latrines were placed across the camp site. All participants, including leaders and service staff lived in tents.

The participants prepared their own meals on gas stoves from supplies provided from the camp supermarkets. Cooking on a gas stove and managing fire posed a risk of burns. The camp staff was provided food in a large mess-tent.

The Swedish weather, with alternating rain and hot sunshine, made proper dressing challenging for participants more used to warmer climates. The daily programme included climbing, chopping and knife-handling, which implied risks of injuries. Furthermore, being an adolescent itself, far from home in a new environment with cultural difficulties, is a psychological challenge. Psychological issues at a camp can range from homesickness to neuropsychological disorders and sometimes behavioural or psychiatric episodes [11].

### Healthcare at campsite

Health care was provided by the Medical Service Section. The planning team consisted of 18 persons from Sweden, Denmark and Germany. Planning of the health care started three years before the camp and was made on a voluntary basis. The Medical Service Section consisted of a section team and three blocks; Health Centre block, Medical Centre block and Emergency Response and Transport block. In general the WSJ was organized with double leadership on all management positions. This secured a robust and flexible organization through the long period of preparation, the intensive period of implementation and during the follow-up period. In the medical section the shared leadership meant that there were two leaders for the section and two leaders for every block. During the executive phase of the camp period the section and block leaders were on call every second day. The remaining members of the planning team also had designated areas of responsibility.

Three Health Centres where placed in the northern, middle and southern part of the camp site. Each Health Centre was formed of three container house modules put in an open formation providing facilities for basic health care. The modules consisted of examination rooms, an office and a supply room. In an outside waiting area patients could rest and wait to be examined. Each Health Centre was staffed with at least one nurse and one physician with a Swedish licence. The Health Centres were staffed during daytime.

The Medical Centre was placed near the Jamboree Head Quarter. The location was chosen in order to have a close proximity with core communication infrastructure and camp decision makers in case of a major crisis. The Medical Centre had extended access to facilities and equipment, prepared for more advanced care. It was equipped with a small laboratory providing urine tests, pregnancy tests, Streptococcus A rapid test and C - reactive protein. The Medical Centre also had bunk beds for patients who were not able to sleep in their tent. Patients staying overnight were taken care of by their own leaders. The Medical Centre provided health care 24 hours per day.

Adjacent to the Medical Centre was a cabin with X-ray facilities and ultrasound. X-ray was mainly used for limbs, the ultrasound for gynaecological examinations by a gynaecologist. The X-ray was open daytime and staffed by specialist nurses from the local hospital. X-ray scans were electronically transferred to the hospital and evaluated by radiologists. A dentistry unit was staffed by a dentist who took care of emergency dental problems.

The camp area was patrolled by first aid personnel who made first assessments when needed. In a medical emergency, radio communication was used through a coordinating emergency control centre that dispatched necessary units. Emergency medical response consisted of ambulances and bicycles. The latter were optimal for fast transportation in crowded areas.

A pharmacy trailer was available in a central area of the camp site and open normal office hours. Prescriptions issued at the camp were handled and delivered by the pharmacy trailer in cooperation with a pharmacy in Kristianstad. By this arrangement drugs prescribed could be delivered every day at the camp. The pharmacy also provided “over the counter drugs” like e.g. painkillers, antihistamines, omeprazol, loperamid, and hydrocortisone cream.

During planning period, cooperation with the local hospital in Kristianstad was established and common routines for points of contact were developed.

The food section of the Jamboree was responsible for staff education about food hygiene and food handling.

## Results

Of total 40,061 scouts, the medical service registered 3,229 (7.3%) visits. Of those 3,229 visits, 2,893 (89.6%) were primary contacts and 336 (10.6%) were follow-up appointments. The morbidity during WSJ 2011 is listed in Table 1. Table 2 lists primary contacts for medical service distributed to the camp role of the patients.

**Table 1:** Morbidity, reason for primary contact with medical service

**Morbidity**	**n=3229**	**Amount related to the total amount of medical contacts %**
Single treatment per patient	2893	89.6
More than one treatment per patient	336	10.4
Reason of primary contact:		
Cut	284	8.8
Fall/Hit	220	6.8
Pinch	24	0.7
Dental	10	0.3
Burn	88	2.7
Fracture/sprain	261	8.1
*Total surgical cases*	*887*	*27.4*
Gastroenteritis	59	1.8
Upper respiratory tract infection	253	7.8
Pneumonia	7	0.2
Eyes	65	2.0
Skin infection	124	3.8
Insect bite	208	6.4
Allergy/urticaria	65	2.0
Dehydration	72	2.2
Headache	72	2.2
Psychological issues	16	0.5
*Total medical cases*	*941*	*28.9*
Unspecified	1065	33.0

**Table 2:** Primary contacts for medical service distributed to the camp role of the patient

	**Total**	**%**
Participants	1847	63.8
Staff members	1023	35.4
Visitors	23	0.8
**Total**	**2893**	

Working in the Medical Section were 153 volunteers from 19 countries (see Table 3). All had different medical background but a previous scouting experience was a common denominator. In addition to being physicians, nurses, counsellors, first aid personnel and assistant nurses, there were a large number of medical students. The professions of the members in the medical section and their legal licence status in Sweden are listed in Table 4. Physicians from several specialties were represented: general practise, orthopaedic surgery, anaesthesiology, internal medicine, gynaecology, paediatrics and neonatology. Of great value for the medical section was also a psychologist specialized in adolescence.

**Table 3:** List of countries represented within the medical section

Austria	Belgium	Brazil	Canada
Czech Republic	Denmark	England	Finland
France	Germany	Italy	Lichtenstein
Luxembourg	Netherland	Spain	Sweden
Switzerland	Tunisia	USA	

**Table 4:** Numbers of medical section staff, their occupation on the camp and their legal licence status in Sweden

Swedish doctors	2
Foreign doctors with Swedish licence	13
Foreign doctors without Swedish licence	4
Swedish nurses	14
Foreign nurses with Swedish licence	8
Foreign nurses without Swedish licence	6
Nurses aid and medical students	30
Other (counsellors, dentist)	9
First aid personnel (mostly medical students)	32
Transport/logistics	17
Planning team members	18
**Total**	**153**

Approximately 400 prescriptions were dispensed by the pharmacy. The pharmacy gave self-care advice to more than 1,000 participants.

128 x-ray examinations were made at the camp. Most of these were x-rays of the limbs, but also a few chest x-rays were performed. Eight participants were sent to the local hospital for other radiologic examinations such as magnetic resonance imaging, computed tomography and non-gynaecological ultrasound.

Two participants had a precondition of chronic kidney failure and needed peritoneal dialysis. Dialysis fluid was provided by the patients and sent to the camp beforehand. A specific area was dedicated for peritoneal dialysis in one of the cabins at the Medical Centre.

During the camp 25 participants with gastroenteritis were reported within a group of scouts. These turned out to be caused by food poisoning. Five participants had to be sent to the local hospital due to dehydration following gastroenteritis. In total the local hospital treated 40 participants as inpatients and 84 participants as outpatients with various diagnoses.

## Discussion

For 12 days a medical service on the level of an outpatient urgent care centre was established for 40,061 participants and staff members forming the 22^nd^ World Scout Jamboree in Sweden. We found an equal distribution of cases to approximately one-third surgical, one-third medical and one-third unspecified cases. The category “unspecified” contains a broad variation of minor cases. They range from minor psychological issues or seeking medical advice to causes that where not indexed. These contacts with the medical section where regularly without a strong medical indication. However, it causes considerable workload on the medical personnel. Of all members of the camp 7.3% needed medical assistance. These numbers are lower than reported by Saeger et al. who found 18.2% of his participants in need of medical assistance during 10 days of camp [12]. The lower number we found can in part be explained that some contingents had their own contingent doctor who took care of minor cases for their own participants. As an example: the Finnish contingent was staffed with two Finnish doctors taking care only of Finnish participants. They registered 219 visits from Finnish participants. The total number of contingents who brought their own doctors to the camp is unknown since it was not mandatory for them to inform the camp management. We had a fairly good overview over the biggest contingents and their medical service since they kept a close communication with the medical section. Hence, the real number of people on the camp that needed medical care during the camp was slightly higher than 7.3 %. However, we can indicate that it was not as high as reported by Saeger et al.

Staff doctors and nurses within the medical section had different areas of expertise and multinational background. Routines were developed to follow good medical practise and Swedish legal requirements. Foreseen common problems were listed in treatment guidelines, e.g. hygiene rules, handling of injection accidents or referral of patients to the local hospital. Swedish guidelines for antibiotic treatment were translated to English. The guidelines were easily available in binders as well as on all computers at the Health Centres and the Medical Centre. The guidelines simplified the familiarizing of foreign health personal to the Swedish standards. It would have been of value to make the guidelines available to the staff before the camp. Due to problems with pre-registration of staff members before the camp that wasn’t possible.

During the camp some new guidelines had to be created and some old guidelines had to be revised by the planning team. To inform the staff of this and other points of interest a newsletter was written every evening and was distributed to the Health Centres and the Medical Centre.

### There were several medical threats identified before the camp

#### Infectious and contagious diseases

A large amount of people on a small area with limited and basic facilities raises the risk of epidemic gastrointestinal disease. Such outbreaks are very difficult to contain [14]. The most effective way to prevent this is hand hygiene and professional food handling [15]. A possible outbreak needs to be registered early and contained. A dedicated staff member monitored gastrointestinal cases during the camp. Action was taken to prevent outbreaks by improving hygiene and isolating single cases. There was no noteworthy outbreak of gastroenteritis at the World Scout Jamboree. This was in our opinion due to good hygiene, and due to good participant and staff education about food storage, hand washing hygiene and facility cleaning.

In Sweden there are less cases of multi resistant bacteria (MRB) compared to other countries in and outside Europe [16]. To prevent spread of MRB from patients originating outside Sweden, admitting routines are in place in Swedish hospitals to screen this population. Screening is also mandatory for medical personnel who have worked in health care units outside of Sweden. During the camp there where more than 40,000 people from all over the world gathered, many from MRB endemic countries. The risk of MRB transmission was high at the camp. It was vital for the planning to establish a good communication between the medical camp organization and the Regional Centre for Communicable Disease Control and Prevention, Skåne County, as well as the local hospital to establish common routines. For logistic reasons the campsite was considered “abroad” in MRB terms. This meant that all referred camp participants or staff members, regardless nationality were treated according to the MRB protocol at the hospital.

In spring of 2011 there were two outbreaks of measles in Europe and an outbreak of Shiga-toxin-producing Escherichia coli in northern Germany [17,18]. These contagious diseases could lead to health problems in an international camp such as ours. To prevent the spread of infectious disease, the Regional Centre for Communicable Disease Control and Prevention, Skåne County, kept the Medical Organization informed and gave advice and recommendations. A close contact with the responsible governmental health institutions is recommended.

#### Psychological issues

In a camp of this size with participants of different cultural and religious background, coping with emotional stressors is challenging for the participants. There will always be a considerable amount of children with psychological issues like anxiety, loss of control, self-harm, depression or drug-related behaviour. These issues are more likely to rise to the surface in an emotionally stressed environment as a summer camp. Special precautions had to be taken to take care of participants and staff members who needed psychological assistance. During camp the psychological and social support was primarily provided by dedicated non-professional counsellors for participants and staff who were called “listening ears”. They could be called by medical staff if needed. Although there were few psychiatric emergencies during the camp, the listening ears supported a considerable number of participants and staff members. Many unspecified contacts with the medical section were related to minor psychological issues. The listening ears were not a part of the medical structure. It is crucial that the camp management understand the importance for psychological support in this camp setting to prevent aggravation of subclinical symptoms. In retrospect the treatment of psychological issues was one of the major challenges the medical section had to handle.

## Conclusions

In summer 2011 a World Scout Jamboree with 40,031 participants and staff members where organized in southern Sweden. The medical section was organized and staffed with members from different nations, with different medical, language and cultural backgrounds. Good preparations with clear guidelines about medical treatment as well as hierarchical structures are important to prevent misunderstandings or suboptimal care. During the camp it is necessary for the leadership to be able to make fast decisions and revise routines. There must also be good ways of communicating decisions to the staff. Different cultural and religious backgrounds of the participant population need to be considered in the planning. Special attention should be paid in prevention of contagious diseases and taking care of psychological issues.

Although the medical structure at the campsite had high competence, the level of treatment was being kept on that of a general practice. Providing more advanced care at the camp, with professionals from different backgrounds and normally not working together, could actually have endangered patient safety. Health care was provided on the camp site in order to reduce the burden on local health care without compromising participants or staff member’s safety and health.

A camp of this size with a multicultural population demands flexible staff with high communication skills.

## Abbreviations

WSJ: World Scout Jamboree; MRB: Multi resistant bacteria.

## Competing interest

The Authors declare that there is no conflict of interest. This research received no specific grant from any funding agency in the public, commercial, or not-for-profit sectors.

## Authors’ contributions

IJ conceived the first draft, analysed the numbers and finalized the manuscript. CAA, ALO, BS and AEW participated in drafting the manuscript. SRH participated in drafting the manuscript and organized the paper handling funding. All authors participated in planning and running the medical section at the World Scout Jamboree. All authors read and approved the final manuscript.

## Pre-publication history

The pre-publication history for this paper can be accessed here:

http://www.biomedcentral.com/1472-6963/13/187/prepub
